# Editorial: Bioinspired nanomaterials: Design principles for imaging and therapeutic

**DOI:** 10.3389/fchem.2022.1079927

**Published:** 2022-11-21

**Authors:** Chun-Pei Shih, Yongdoo Choi, Chen-Sheng Yeh, Peilin Chen

**Affiliations:** ^1^ Research Center for Applied Sciences, Academia Sinica, Taipei, Taiwan; ^2^ Division of Translational Science, National Cancer Center, Goyang-si, South Korea; ^3^ Department of Chemistry, National Cheng Kung University, Tainan, Taiwan; ^4^ Institute of Physics, Academia Sinica, Taipei, Taiwan

**Keywords:** nanomaterial, imaging, therapeutic, Nanoparticle (NP), bioinspired

Over the last few decades, the advancements in nanotechnology have prompted developments in various fields of research, especially in the area of biomedical science. For example, many research groups have extensively studied the application of nanomaterials for imaging and therapeutic. To probe the complex biological environment of the human body, it is essential for nanomaterials to possess the necessary properties to deal with different situations and yield various valuable information for in-depth analysis. The development of bioinspired nanomaterials brought about numerous advantages such as traversing the circulatory system while avoiding immune responses or achieving specific targeting of disease sites. In this editorial, we will briefly summarize the findings and discussions that were presented in each of the accepted articles.

We start off with a mini-review article by Shih et al. that summarize the design principles of bioinspired interfaces. Several discussions were made in the mini-review including recent progress of different cell membrane coatings for nanoparticle (NP), surface modification techniques for NPs, and the functionalization of NPs with geometric property variations. It should be noted that most discussions were made based on imaging and therapeutic applications. In the last part of the mini-review, future perspectives for NP design were elaborated to provide the readers with key ideas for further developments in this research field.

In the review article by Vincy et al., comprehensive discussions were made regarding red blood cell-derived particles. Synthetic strategies, characterization methodology, and applications were enumerated in detail. In addition, the review article also provides key insight into the current state of clinical translation as well as the challenges that it may face during the transition from lab to clinical applications.

Due to the growing interest in NP-related applications, much research focusing on various aspects of the field of NP study has surfaced over the years. Therefore, we include three original research articles that describe interesting therapeutic applications of NPs, such as inhibition of cancer cell proliferation or improvement in drug delivery efficiency.

Mochizuki et al. proposed an X-ray sensitizer that was based on the surface functionalization of organosilica NPs with sub-nm-sized gold NPs. The synthesized NPs were further modified with polyethyleneimine and tested against 4T1 mouse mammary tumor cells. As shown in the result, the tumor cells displayed significant inhibition of cell proliferation and an increase in cell death by X-ray irradiation at 8Gy after being treated with the synthesized NP. Furthermore, it was demonstrated that the NP can act as an oxidant, which can induce DNA damage by mitochondrial deactivation. Thus promoting non-apoptotic cell death *via* radiotherapy.

In another study, Chen et al. investigated the effect of surface charge and size dependency of mesoporous silica nanoparticles (MSNs) for penetrating the blood-brain barrier (BBB). They discovered that the ideal condition for MSN to permeate the BBB in larval zebrafish was negative surface charged and small particle size (50 nm). The potential applications for brain disease treatment were shown when they conducted the test with doxorubicin-loaded MSNs and obtained promising results.

For most NP-related studies in cancer therapy, NPs were often subjected to the enhanced permeation and retention (EPR) effect for tumor uptake. In order to exploit this property, NPs were often designed to reach lower size limits. However, in the work by Chen et al., they have demonstrated that the vessel permeability of NPs at the lower size limit was found to be dependent on the mouse strain and surface charge of the NPs. This work could provide valuable insights to other research groups if anionic NPs were employed in their study.

In order to provide a more macroscopic view of the applications of nanomaterials, we include original research articles that describe fluorescence imaging of extracellular potassium ions and novel antimicrobial surface coating materials. We hope that by including these articles, the readers will have a better understanding of various kinds of nanomaterials that can be applied in different situations.

The fluorescence imaging technique involving the use of a conjugate of a quadruplex structure-forming oligonucleotide with a peptide incorporating a Förster Resonance Energy Transfer (FRET) chromophore pair was proposed by (Sato et al.). The potassium-sensing oligonucleotide enables real-time imaging of cytoplasmic potassium ion concentrations, which could be an important indicator when monitoring potassium ion concentration *in vivo*.

Ayalew et al. developed a novel electroactive antimicrobial surface coating that possesses powerful bactericidal efficiency (∼92% bacterial death) and enhanced biocompatibility with mammalian cells. They further modified the polymer to enhance the protein adsorption and mammalian/bacterial cell binding resistance capabilities, thus producing a copolymer with both antimicrobial and antifouling properties.

The future of nanotechnology is moving towards the development of nanomaterials with multiple integrated functions which could expand the range of applications. For instance, the development of hybrid NPs enables imaging and therapeutic functions simultaneously (Hwang et al., 2021; Chen et al., 2022). There is also significant progress in the field of nanodevices, such as the integration of nanomaterials with different platforms such as organic electrochemical transistors (Janardhanan, et al., 2022) or microfluidic (Hsiao et al., 2022). We hope that this Research Topic would help the readers to have a better grasp of the concept of bioinspired nanomaterials as well as the roles that they play when it comes to the applications and future developments for imaging and therapeutics.
